# Twenty Years of Active Bacterial Core Surveillance

**DOI:** 10.3201/eid2109.141333

**Published:** 2015-09

**Authors:** Gayle Langley, William Schaffner, Monica M. Farley, Ruth Lynfield, Nancy M. Bennett, Arthur Reingold, Ann Thomas, Lee H. Harrison, Megin Nichols, Susan Petit, Lisa Miller, Matthew R. Moore, Stephanie J. Schrag, Fernanda C. Lessa, Tami H. Skoff, Jessica R. MacNeil, Elizabeth C. Briere, Emily J. Weston, Chris Van Beneden

**Affiliations:** Centers for Disease Control and Prevention, Atlanta, Georgia, USA (G. Langley, M.R. Moore, S.J. Schrag, F.C. Lessa, T.H. Skoff, J.R. MacNeil, E.C. Briere, E.J. Weston, C. Van Beneden);; Vanderbilt University School of Medicine, Nashville, Tennessee, USA (W. Schaffner);; Emory University School of Medicine and The Atlanta VA Medical Center, Atlanta (M.M. Farley);; Minnesota Department of Health, St. Paul, Minnesota, USA (R. Lynfield);; University of Rochester, Rochester, New York, USA (N.M. Bennett);; University of California, Berkley, California, USA (A. Reingold);; Oregon Department of Human Services, Portland, Oregon, USA (A. Thomas);; Johns Hopkins Bloomberg School of Public Health, Baltimore, Maryland, USA (L.H. Harrison);; New Mexico Department of Health, Santa Fe, New Mexico, USA (M. Nichols);; Connecticut Department of Public Health, Hartford, Connecticut, USA (S. Petit);; Colorado Department of Public Health and Environment, Denver, Colorado, USA (L. Miller)

**Keywords:** Emerging Infections Program, surveillance, invasive bacterial infections, EIP, Active Bacterial Core Surveillance, ABCs, bacteria

## Abstract

Active Bacterial Core surveillance (ABCs) was established in 1995 as part of the Centers for Disease Control and Prevention Emerging Infections Program (EIP) network to assess the extent of invasive bacterial infections of public health importance. ABCs is distinctive among surveillance systems because of its large, population-based, geographically diverse catchment area; active laboratory-based identification of cases to ensure complete case capture; detailed collection of epidemiologic information paired with laboratory isolates; infrastructure that allows for more in-depth investigations; and sustained commitment of public health, academic, and clinical partners to maintain the system. ABCs has directly affected public health policies and practices through the development and evaluation of vaccines and other prevention strategies, the monitoring of antimicrobial drug resistance, and the response to public health emergencies and other emerging infections.

## Abstract 2

This program has directly affected public health policies and practices.

Active Bacterial Core surveillance (ABCs), a program in the Centers for Disease Control and Prevention (CDC) Emerging Infections Program (EIP) network, was launched in 1995 as part of the CDC strategy to address the worldwide threat of emerging infectious diseases (*1*). The goals of EIP are to detect and investigate emerging pathogens; integrate laboratory science and epidemiology; enhance communication about emerging diseases; and strengthen the state and federal public health infrastructure with regard to surveillance, prevention and control programs. Before establishment of EIP, little was known about the national burden of many of the disease areas now under its surveillance umbrella, which include foodborne diseases, influenza-related hospitalizations, health care–associated infections, and invasive bacterial infections.

ABCs and other EIP activities are collaborations between CDC, state and local health departments, and academic institutions. Originally established at 4 sites (California, Connecticut, Oregon, and Minnesota), by 2003 ABCs added Georgia, Maryland, New York, Tennessee, Colorado, and New Mexico, for a total of 10 sites. The sites represent geographic diversity and approximate the racial composition of the US population (*2*). Currently, the population under surveillance ranges from 19 to 42 million (up to 12% of the US population), depending on the pathogen.

ABCs provides population-based surveillance for select causes of invasive bacterial infections in the community, primarily manifested as bloodstream infections and meningitis. At its inception and continuing today, it includes surveillance for invasive infections caused by group A *Streptococcus* (GAS), *Haemophilus influenzae*, *Neisseria meningitidis*, group B *Streptococcus* (GBS), and *Streptococcus pneumoniae*. Surveillance for invasive methicillin-resistant *Staphylococcus aureus* (MRSA), which had long been recognized as a significant nosocomial pathogen, was added to surveillance in 2004 because it had emerged as a substantial cause of invasive infections in the community (*3*). In 2001, rising rates of pertussis (http://www.cdc.gov/pertussis/surv-reporting.html) and legionellosis (*4*) led to the addition of special surveillance for these diseases to ABCs.

Invasive pneumococcal disease (IPD) provides an example of the power of this large, sustained, population-based surveillance system for evaluating public health interventions and providing feedback for additional prevention measures. Although *S. pneumoniae* is a major cause of invasive infections (e.g., bloodstream infections and meningitis) in the United States and worldwide, IPD is not reportable in all states. In the late 1990s, when a new vaccine was being developed, a system was needed to determine baseline rates of IPD, evaluate vaccine effectiveness, and monitor circulating serotypes. After establishment of ABCs to fill the void of tracking the disease burden of IPD and other major causes of invasive bacterial disease in the United States, ABCs detected a large reduction in IPD detected among children <5 years of age (for whom vaccination with 7-valent pneumococcal conjugate vaccine [PCV7] was recommended in 2000) and among adults, who benefited from herd protection. ABCs also recognized an increase in IPD rates caused by *S. pneumoniae* serotypes absent from PCV7; this information resulted in accelerated approval of a 13-valent pneumococcal vaccine (PCV13) that has resulted in further reductions in IPD.

Not all infections captured under ABCs are reportable to CDC. Even for those infections included in the CDC National Notifiable Disease Surveillance System, case counts may be underestimated because they rely on reporting by laboratories and clinicians, whereas ABCs tries to actively identify 100% of the cases within the surveillance area. Additionally, epidemiologic data collected by health departments are often incomplete because of limited resources and the inflexibility of the system to capture variables of interest. Unlike the National Notifiable Disease Surveillance System, ABCs also collects isolates and serotypes and tests them for antimicrobial drug susceptibility**.** These attributes enable ABCs to fulfill 2 critical objectives: 1) to determine the incidence and epidemiologic characteristics of invasive diseases under surveillance and 2) to determine molecular epidemiologic patterns and microbiological characteristics of these invasive infections.

## ABCs Methods

For routine surveillance, a case of invasive bacterial disease is defined as isolation of *H. influenzae, N. meningitidis*, GAS, GBS, *S. pneumoniae*, or MRSA from a normally sterile body site (e.g., blood, joint, pleural, or cerebrospinal fluid) in a resident of the surveillance area. Additionally, cases include ill persons from whom GAS is isolated from a wound or other tissue in the presence of necrotizing fasciitis or streptococcal toxic shock syndrome. Cases of GBS in a mother are also included if GBS has been isolated from the placenta or amniotic fluid in the event of fetal death.

The ABCs approach to surveillance is distinctive; it is active, laboratory based, and population based. The goal is to detect 100% of laboratory-confirmed cases by actively contacting all clinical laboratories that routinely process specimens from residents of the surveillance area. Audits are performed regularly to ensure case capture. Efforts at most sites to make ABCs pathogens reportable to state public health agencies have facilitated participation of almost all laboratories (≈600) that serve the surveillance population. Because the population under surveillance is well-defined, US Census data are used to calculate disease incidence rates within the ABCs population. Because of the large population base, CDC uses ABCs data to estimate the national disease burden after adjusting for race and age distribution in the United States.

Medical records review is used to collect demographics, clinical course, outcome, infection type, underlying conditions, and vaccination history for each case-patient. For most patients, an isolate from the first positive culture is collected. Since 1995, with the exception of MRSA (for which a convenience sample of 100 isolates has been collected since 2005), ≈85% of isolates have been collected from eligible patients. Isolates undergo serologic or molecular typing and standardized antimicrobial drug susceptibility testing at CDC or other reference laboratories. A collection of ≈80,000 *S. pneumoniae*, GAS, GBS, *N. meningitides*, and *H. influenzae* isolates is accessible to ABCs partners and external researchers by request (http://www.cdc.gov/abcs/pathogens/isolatebank/index.html). ABCs MRSA isolates are deposited at the Network on Antimicrobial Resistance in *Staphylococcus aureus,* a repository sponsored by the National Institute of Allergy and Infectious Diseases at the National Institutes of Health (http://www.niaid.nih.gov/labsandresources/resources/dmid/narsa/Pages/default.aspx).

The ABCs infrastructure is also used to conduct surveillance for other bacterial diseases and provides a foundation for epidemiologic investigations. Examples include special surveillance for pertussis and legionellosis, case–control studies to assess vaccine effectiveness, and cohort studies to assess the uptake and effectiveness of other public health interventions.

## ABCs Effects on Vaccine Development, Evaluation, and Policy Recommendations

Because of the large, representative catchment area and the laboratory-linked, population-based epidemiologic data, results from ABCs have been used in the development and prelicensure evaluation of multiple vaccines. After licensure, ABCs data have been used to formulate policy recommendations and to determine the real-world impact of vaccines (Table 1).

**Table 1:** Key uses and findings of Active Bacterial Core surveillance data for vaccine development, evaluation, and policy formulation*

Pathogen	Vaccines	Key uses and findings
*Streptococcus pneumoniae*	PCV7 and PCV13	Selection of serotypes included in PCV7and PCV13
		Informed ACIP recommendations for children <5 y of age
		Tracking postlicensure declines in cases
		Documented effectiveness of PCV7
		Monitoring incidence of nonvaccine serotypes
		Accelerated regulatory approval of PCV13
		Informed ACIP recommendations for PCV13 use in immunocompromised adults and children
*Neisseria meningitidis*	Conjugate vaccines, serogroup B vaccines	Informed ACIP recommendations for children 11–18 y of age
		Informed ACIP recommendations for booster dose
		Documented vaccine effectiveness
		Informed ACIP infant meningococcal recommendations
		Evaluated potential effect on serogroup B disease in United States
*Haemophilus influenzae*	Hib vaccine	Tracking postlicensure declines in Hib disease
		Tracking shift toward non-Hib disease;
		Evaluated effect of vaccine shortages
Group A *Streptococcus*	M-type vaccine (under development)	Estimated degrees of protection against severe group A streptococcal infections
Group B *Streptococcus*	Trivalent vaccine (under development)	Informing development of vaccine to prevent early-onset (within 1 week of life) group B streptococcal disease
Methicillin-resistant *Staphylococcus aureus*	*S. aureus* vaccine (under development)	Determining population groups to target

*ACIP, Advisory Committee on Immunization Practices; Hib, *H. influenzae* type b vaccine; PCV7, 7-valent pneumococcal conjugate vaccine; PCV13, 13-valent pneumococcal conjugate vaccine. An expanded version of this table with references is available in the online Technical Appendix (http://wwwnc.cdc.gov/EID/article/21/9/14-1333-Techapp1.pdf).

**Figure 1:**
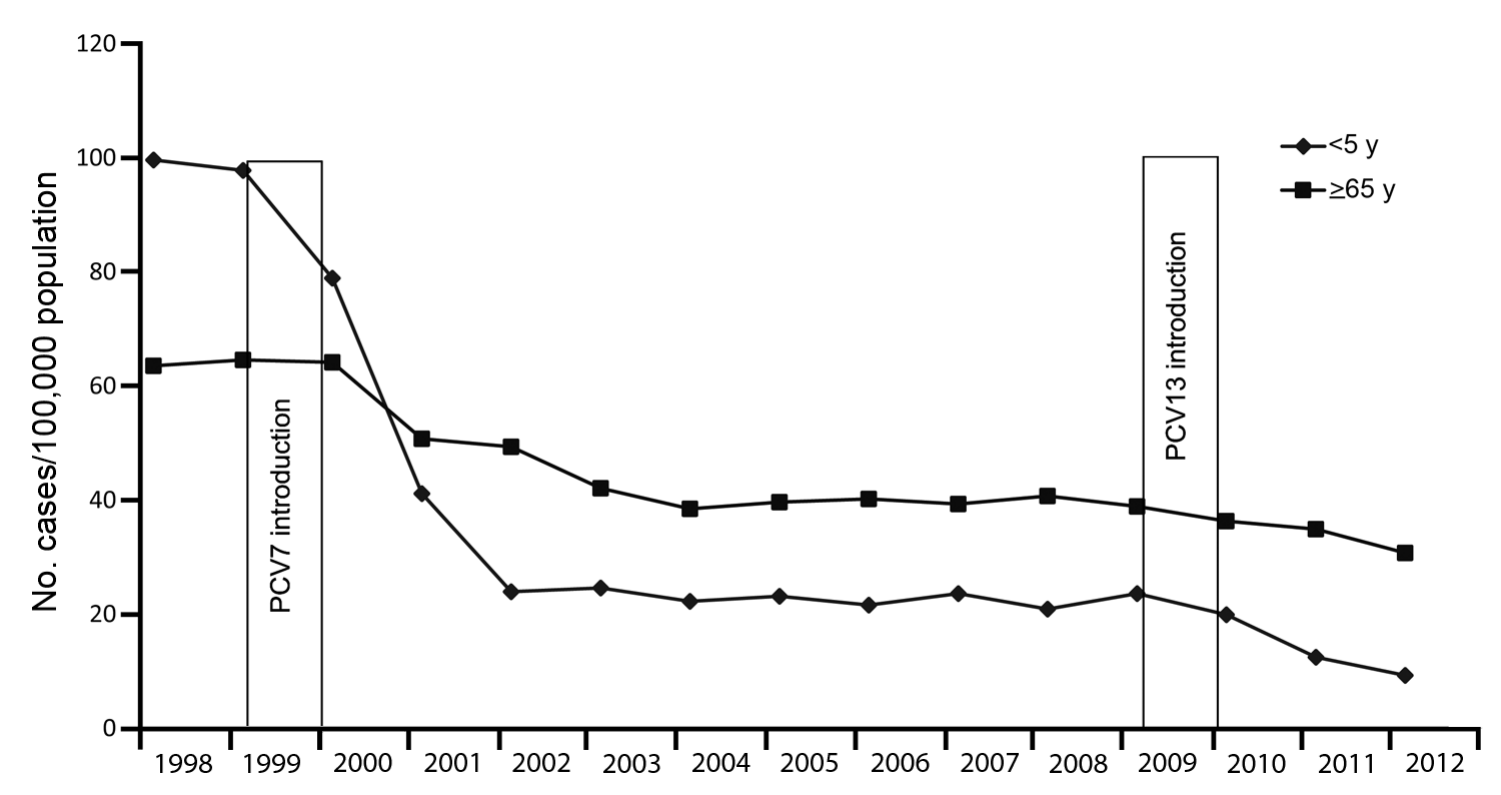
Incidence of invasive pneumococcal disease in children <5 and adults >65 years of age, Active Bacterial Core surveillance, United States, 1998–2012. PCV7, 7-valent pneumococcal conjugate vaccine; PCV13, 13-valent pneumococcal conjugate vaccine.

As mentioned earlier, ABCs closely tracked the decline in IPD in children after the introduction of PCV7 (Figure 1). Perhaps a more surprising finding, which would not have been possible without the large ABCs catchment area that includes surveillance among all age groups, was the decline in vaccine-type IPD among adults, particularly those >65 years of age (Figure 1). ABCs also identified increased incidence of IPD for serotypes not found in PCV7; particularly serotype 19A. These findings contributed to the accelerated approval of PCV13, which includes serotype 19A, and the recommendation for its use in children <5 years of age. Rates of IPD have further declined since introduction of PCV13 (Figure 1).

Age- and serogroup-specific ABCs data highlighted the increased risk for vaccine-preventable meningococcal disease among college students, adolescents, and young adults. These findings contributed to the Advisory Committee on Immunization Practices policy recommendation for routine use of meningococcal conjugate vaccines in all persons 11–18 years of age and subsequent recommendations for a booster dose during late adolescence.

**Figure 2:**
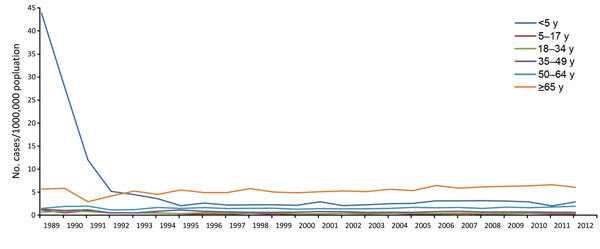
Incidence of invasive *Haemophilus influenzae* disease, by age group, United States, 1989–2012.

Through long-standing surveillance, ABCs was able to document the persistent decline of invasive *H. influenzae* infections among young children after introduction of type b vaccine in the mid-1980s (Figure 2). ABCs surveillance for *H. influenzae* type b (Hib) disease was critical for monitoring how vaccine shortages affected disease rates. Because of the availability of epidemiologic data linked to serotype determination, a shift toward non-Hib disease in adults in the post–Hib vaccine era has been recognized.

Although trend data may show indirect evidence of a vaccine’s effectiveness, proof of effectiveness requires a more formal epidemiologic investigation to account for other factors that may influence the decline in disease incidence. The ABCs infrastructure was used to conduct case–control studies that confirmed the effectiveness of conjugate meningococcal and pneumococcal vaccines against invasive disease. These very large studies could only have been done through an integrated network, and they highlight the efficiencies gained by maintaining such an infrastructure.

Serotype and serogroup data from ABCs pathogens are also being used to help with formulation of vaccines and evaluation of the potential effectiveness of vaccines currently under development, including those products targeting GAS, GBS, *S. aureus*, and serogroup B meningococcal disease. ABCs data have been used to predict the effectiveness in the United States of a 26-valent GAS vaccine and now a 30-valent GAS vaccine that is under development. GBS disease burden and serotype data gathered through ABCs have been used to inform development of a trivalent GBS vaccine now in phase I and II trials. ABCs data have been used to determine which population groups would be the best candidates for receipt of *S. aureus* vaccines to prevent invasive MRSA disease and to evaluate the potential effect of serogroup B meningococcal vaccines on disease burden in the United States.

## ABCs Effect on Other Prevention-Related Policies and Practices

**Figure 3:**
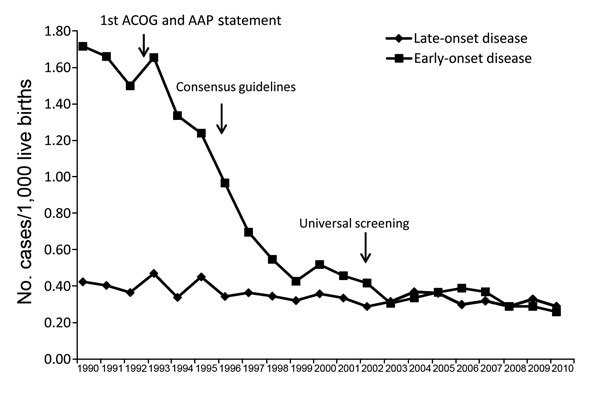
Incidence of early-onset group B *Streptococcus* disease before and after issuance of guidelines, United States, 1990–2010. AAP, American Academy of Pediatrics; ACOG, American Congress of Obstetricians and Gynecologists.

ABCs and a precursor surveillance system for GBS were used to define the need for guidelines for providing antimicrobial drugs to pregnant women during delivery to prevent early-onset GBS in their newborns; such guidelines were published in 1992 (*5*,*6*) and 1996 (*7*). Without evidence as to which strategy was better, the 1996 guidelines recommended that health care providers could use either a screening or risk-based approach to decide which women should receive prophylaxis during delivery. An ABCs-based cohort study that sampled from a population of ≈600,000 live-born infants at 8 sites demonstrated the value of screening over the risk-based approach. Specifically, universal prenatal screening of pregnant women for vaginal/rectal colonization with GBS and providing antimicrobial drugs during delivery to those who were colonized was ≈50% more effective at preventing early onset GBS than providing prophylaxis to pregnant women on the basis of certain risk factors (*8*). This finding led to issuance of new guidelines in 2002 (*9*) and revised guidelines in 2010 (*10*), which resulted in further reductions in disease. Since the early 1990s, ABCs has documented a >80% decline in the incidence of early-onset GBS infection and prevention of an estimated 70,000 cases of early-onset GBS infection (Figure 3).

Guidelines for the prevention of invasive GAS infections were also informed by ABCs surveillance and special studies. An ABCs study found an increased risk for severe GAS infection among household contacts of index patients (*11*). These data, coupled with data that were collected from routine surveillance on the frequency of GAS infection in postpartum women (*12*) and postsurgical patients provided the foundation for the development of CDC policy guidance in households and health care settings (*13*). ABCs surveillance data on the risk for GAS infections among long-term care facility patients also helped inform prevention and control strategies for those settings (*14*,*15*).

## Monitoring of Antimicrobial Drug Resistance

**Figure 4:**
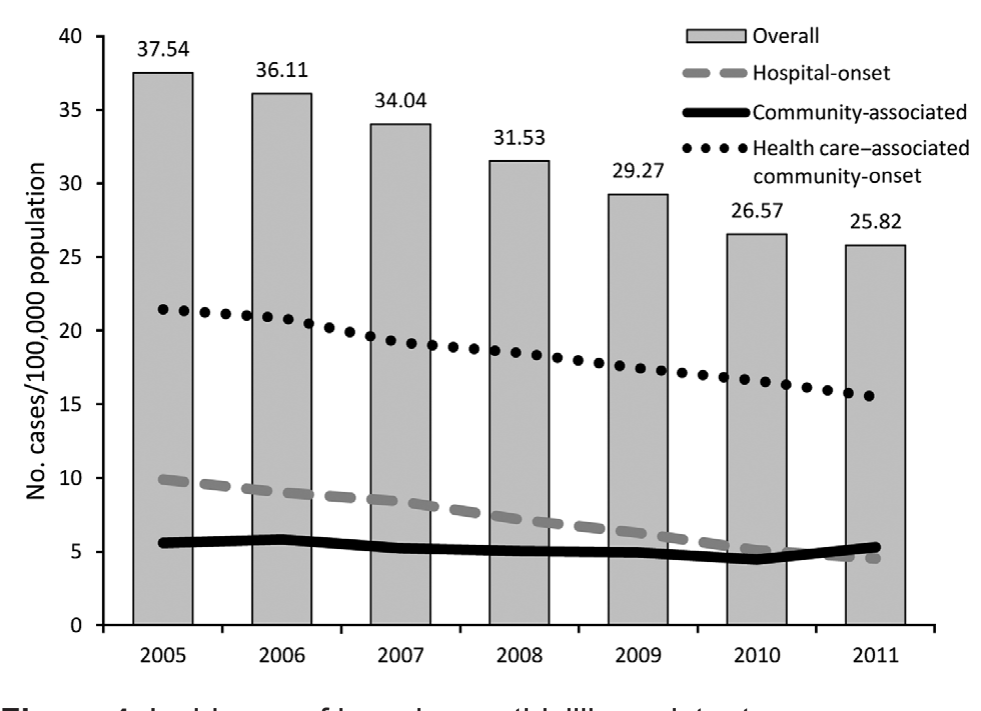
Incidence of invasive methicillin-resistant *Staphylococcus aureus* (MRSA) (defined as MRSA isolated from a normally sterile source) infections, by epidemiologic category, Active Bacterial Core surveillance, United States, 2005–2011 (*20*).

The first nationwide estimates of the burden of invasive MRSA were derived from ABCs; in 2005, ≈94,000 cases and ≈18,000 deaths were attributed to invasive MRSA (*16*). Most (≈84%) infections were health care–associated—either hospital-onset (culture obtained >3 days after admission) or health care–associated community-onset (culture obtained from outpatient or within 3 calendar days after admission from a patient with a health care–associated risk factor, which include presence of a central venous catheter within 2 days before MRSA culture or history of surgery, hospitalization, dialysis, or residence of long-term care facility in the 12 months preceding culture date). The prominence of health care–associated community-onset infections was newly brought to light by the ABCs network (*16*). This report led to increased awareness of MRSA infections, and prevention of health care–associated MRSA became a goal for public health agencies and policy makers (*17*–*19*). ABCs documented a 54% decline in hospital-onset MRSA and a 28% decline in health care–associated community-onset MRSA invasive infections during 2005–2011 (Figure 4) (*20*). An ABCs-based study evaluating risk factors for health care–associated community-onset MRSA infections has just been completed. Despite great progress in reducing health care–associated MRSA infections, rates of invasive MRSA infections in the community among persons without recent health care exposures (community-associated infections) remain largely unchanged, indicating the ongoing need for prevention strategies outside hospital settings (Figure 4) (*20*).

ABCs data showed that, from 1995 through 1998, a large and increasing proportion (up to 25%) of isolates from patients with IPD were resistant to penicillin (*21*). After introduction of PCV7, analysis of ≈43,000 isolates collected from all ABCs sites found a 64% decline in penicillin-nonsusceptible IPD among children <5 years of age and a 45% decline among adults >65 years from 1998–1999 through 2008. This finding demonstrated the effectiveness of routine use of pneumococcal conjugate vaccine in children for reducing the spread of resistant strains on a national scale in all age groups (*22*). However, 30% of ABCs isolates from patients with IPD remain resistant to >1 antimicrobial drug (http://www.cdc.gov/drugresistance/threat-report-2013/).

In contrast to IPD, GAS infections remain sensitive to penicillin. GAS isolates are collected to monitor the resistance of invasive GAS infections to not only β-lactams but also macrolides and other antimicrobial drugs. ABCs data have documented increasing resistance to erythromycin (currently 8%–11%), a macrolide commonly used for treating pharyngitis in children who are allergic to penicillin (http://www.cdc.gov/drugresistance/threat-report-2013/).

Monitoring antimicrobial drug resistance of GBS across a large geographic area is critical because antimicrobial prophylaxis is widely used to prevent early-onset GBS. GBS isolates from ABCs have remained largely susceptible to first-line prophylaxis and treatment with β-lactams, but for some isolates, β-lactam MICs have been increasing (*23*). Increasing resistance of GBS isolates to clindamycin discovered through ABCs prompted a 2002 change in the second-line prophylaxis recommendation for intrapartum women, from clindamycin to cefazolin for penicillin-allergic women at low risk for anaphylaxis (*9*). Two reports of vancomycin-resistant GBS isolates (1 inside and 1 outside the ABCs catchment area) have recently been published (*24*). Although apparently not widespread, vancomycin resistance is a concerning development that must be closely monitored because it is an alternative agent for prophylaxis in penicillin-allergic patients at high risk for anaphylaxis (*10*).

During 2007–2008, ciprofloxacin-resistant *N. meningitidis* was identified in 3 patients: 2 from Minnesota (within the ABCs catchment area) and 1 from a bordering area of North Dakota (not an ABCs site) (*25*). Although *N. meningitidis* isolates had routinely been collected for serogrouping, resistance testing was not routinely done because previous evaluations had shown low levels of antimicrobial drug resistance (*26*). When the potential problem arose with ciprofloxacin, a commonly used agent for prophylaxis of close contacts, the existing ABCs infrastructure was used to test *N. meningitidis* isolates for antimicrobial drug resistance. No additional ABCs isolates collected during 2007–2011 were found to be resistant to ciprofloxacin, providing reassurance that the chemoprophylaxis policy recommendations continued to be sound (*27*).

## Response to Public Health Emergencies and Surveillance for Other Emerging Infections

One of the key attributes of ABCs and other EIP activities is flexibility for responding to public health emergencies. After the 2001 anthrax attack, the ABCs infrastructure was used to establish and test a more sensitive and timely system for identifying inhalation anthrax in Connecticut (*28*). After the discovery of severe acute respiratory syndrome in 2002, the EIP infrastructure assisted with surveillance activities and the investigation of suspected cases (*29*). During the 2009 influenza A(H1N1) pandemic, early recognition of IPD among pandemic influenza patients at the Colorado ABCs site led to increased emphasis on pneumococcal disease prevention strategies (*30*). The Tennessee ABCs site helped the Tennessee Department of Health investigate the recent outbreak of fungal meningitis (*31*).

**Figure 5:**
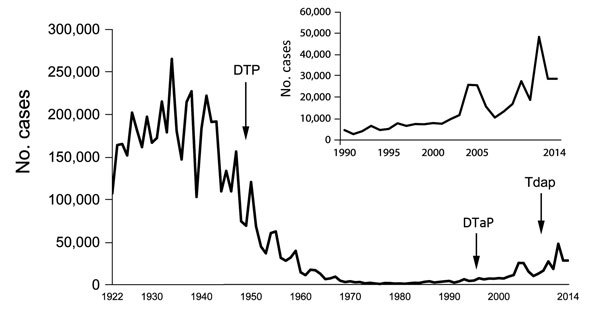
Number of pertussis cases reported to the National Notifiable Diseases Surveillance System, 1922–2014. Inset shows detail view of data for 1990–2014. Sources: Centers for Disease Control and Prevention; National Notifiable Diseases Surveillance System and Supplemental Pertussis Surveillance System, 1922–1949; passive reports to the Public Health Service. Data for 2014 are provisional. DTP, diphtheria, tetanus, pertussis vaccine; DTap, diphtheria, tetanus, acellular pertussis vaccine given to children up to 7 years of age; Tdap, tetanus, diphtheria, acellular pertussis vaccine given to adolescents and adults.

ABCs has also been used as a surveillance platform for other emerging infections, including pertussis and legionellosis. Since the 1980s, the number of reported pertussis cases has been gradually increasing: the 48,277 cases reported in 2012 represent the largest number of cases since 1955 (Figure 5) (http://www.cdc.gov/pertussis/downloads/pertuss-surv-report-2012.pdf). From 2000 through 2009, the age-adjusted incidence of legionellosis has almost tripled, from 0.40 to 1.08 cases per 100,000 persons (*4*). Since 2011, enhanced pertussis surveillance has been conducted at 6 sites and legionellosis surveillance at 10 sites. In addition to enhanced surveillance, a study to estimate the effectiveness of maternal vaccination at preventing infant pertussis is under way.

## ABCs Effect on Domestic and International Surveillance Programs

A goal of ABCs is to share its methods and experiences with domestic and international partners. In addition to providing materials, methods, and results through its website (http://www.cdc.gov/abcs/index.html), outreach to partners has been provided at multiple national and international conferences. ABCs closely collaborated with the South Africa National Institute for Communicable Diseases in the establishment of a similar surveillance system in that country and in sharing lessons learned and epidemiologic findings (http://www.nicd.ac.za/?page=homepage&id=125). ABCs has also been used as the standard for evaluating and validating less expensive methods for tracking antimicrobial drug susceptibility and measuring vaccine effectiveness—methods that can be used in settings with fewer resources (*32*,*33*).

## Challenges and Opportunities

When ABCs began, most cases were identified by reviewing paper laboratory log sheets and computer printouts and most case report forms were abstracted from paper records. The increasing availability of electronic laboratory and medical records may improve timeliness, completeness, and accuracy of reporting (*34*). Ensuring that cases are appropriately captured requires an understanding of laboratory information system codes and periodic reviews of how data are imported. Extracting information and transferring it into usable formats remains a challenge (*35*).

With the exception of surveillance for pertussis and legionellosis, the current ABCs case definition includes only culture-proven disease. Although culture remains the standard for diagnosing invasive infections, the use of culture-independent diagnostic tests will probably increase. Validation of culture-independent diagnostics will remain a major consideration for determining whether culture-independent tests are added to the ABCs case definition.

The growing fields of microbial and human genomics provide ABCs with a potential new role in increasing the understanding of disease transmission and pathogenesis. ABCs is uniquely poised to evaluate the relationship between human and pathogen genetic variation and infectious disease, given that surveillance is population based and that bacterial isolates are collected. A study currently under way is using whole-genome sequencing to compare isolates from patients with GAS and necrotizing fasciitis or streptococcal toxic shock syndrome with isolates from persons with isolated bacteremia; another study is planned to examine potential differences in host genomic factors. ABCs is also validating the use of whole-genome sequencing for outbreaks caused by *N. meningitidis*.

In the United States, the leading cause of illness and death is chronic disease. A better understanding of the associations and interactions between chronic diseases and invasive bacterial infections is needed for a better understanding of the pathophysiology, potential interventions, and prognoses for invasive bacterial infections. ABCs surveillance data coupled with other data sources have been used to analyze the influence of chronic diseases on IPD (*36*) and *H. influenzae* infection in adults (*37*). Efforts to analyze the effects of obesity and diabetes on the incidence and severity of ABCs pathogens are under way.

A major goal of ABCs is assessment of public health disparities and promotion of health equity across population groups. ABCs has documented differences in rates of disease across persons of different races; invasive GBS (http://www.cdc.gov/abcs/reports-findings/survreports/gbs12.pdf), pneumococcal (*38*), and MRSA (http://www.cdc.gov/abcs/reports-findings/survreports/mrsa12.pdf) infections are more common among black than white persons. However, racial differences are just one measure of disparity, and categorizing a person’s race is becoming increasingly difficult as the United States becomes more multiracial. ABCs analyses have incorporated census tract data to determine the association between area-level poverty and disease incidence (*39*,*40*). In 2013, ABCs started incorporating census tract information into routine surveillance.

## Conclusions

ABCs is distinctive among public health surveillance systems in that it is designed to capture nearly all cases of culture-confirmed invasive bacterial diseases over a large, well-defined, and geographically diverse area of the United States. These comprehensive data enable accurate estimations of the national disease burden for severe bacterial infections under surveillance. The collection of isolates in conjunction with epidemiologic data has contributed to the microbiological and molecular characterization of pathogens, which has played a part in the development of vaccines and monitoring of antimicrobial drug resistance. Although the surveillance system alone provides powerful data for informing public health actions, the large ABCs infrastructure provides an efficient and effective platform for engaging in special investigations that would otherwise require additional resources. The infrastructure also provides the flexibility needed to respond to emergencies and to serve as a surveillance platform for other emerging pathogens. Perhaps the greatest strength of ABCs and the reason for its success have been the committed, genial, and long-lasting collaboration among local, state, and federal agencies, academic institutions, and clinical laboratories.

Although ABCs has addressed many questions of public health significance that have directly affected public health policy and practice, many questions still remain (Table 2). To maintain the ability of ABCs to answer questions of high importance, the network must continue to embrace and adapt to the changing public health, laboratory, information technology and medical landscapes.

**Table 2:** Questions left unanswered with regard to Active Bacterial Core surveillance*

Organism or disease	Questions
*Streptococcus pneumoniae*	Should PCV13 be recommended for adults?
	What proportion of invasive pneumococcal disease is preventable with vaccine?
	What other strategies are available to prevent non–vaccine type disease?
*Neisseria meningitidis*	Should serogroup B vaccines be recommended for routine use in the United States?
*Haemophilus influenzae*	Are control strategies (e.g., chemoprophylaxis, vaccines) needed for non-Hib disease?
Group B *Streptococcus*	Will antimicrobial drug resistance reduce the effectiveness of intrapartum prophylaxis?
	What will be the projected effect of vaccines on infant disease?
	Are there interventions to reduce infant late-onset disease?
Group A *Streptococcus*	What age groups should be targeted for vaccines according to potential effect on invasive disease?
MRSA	Can modifiable risk factors for HACO MRSA be identified?
	What are effective strategies for preventing infections outside acute-care settings?
Pertussis	Does the acellular vaccine given during pregnancy effectively prevent pertussis in infants?
	What is the effect of newly emerging *Bordatella pertussis* strain changes on disease epidemiology, clinical presentation, and vaccine effectiveness?
Legionellosis	Why are rates higher among black than white persons and higher among men than women?
	Why do rates differ by geographic area?

*PCV13, 13-valent pneumococcal conjugate vaccine; Hib, *H. influenzae* type b; HACO, health care–associated community onset; MRSA, methicillin-resistant *Staphylococcus aureus*.

Technical Appendix Expanded version of Table 1 (Key uses and findings of Active Bacterial Core surveillance data for vaccine development, evaluation, and policy formulation).
