# Supplementary material for: Twenty Years of Active Bacterial Core Surveillance
Source: Emerg Infect Dis. 2015 Sep;21(9):1520–8. doi: 10.3201/eid2109.141333 (PMC4550139; doi:10.3201/eid2109.141333)
Supplement: Technical Appendix — Expanded version of Table 1 (Key uses and findings of Active Bacterial Core surveillance data for vaccine development, evaluation, and policy formulation). [file 14-1333-Techapp-s1.pdf]

Article DOI: http://dx.doi.org/10.3201/eid2109.141333

Twenty Years of Active Bacterial Core Surveillance

Technical Appendix

Technical Appendix Table. Key uses and findings of Active Bacterial Core surveillance data for vaccine development, evaluation, and policy formulation*

Pathogen	Vaccines	Key uses and findings
Streptococcus pneumoniae	PCV7 and PCV13	Selection of serotypes included in PCV7and PCV13<br>Informed ACIP recommendations for children <5 y of age (*1,2*)<br>Tracking postlicensure declines in cases (*3*)<br>Documented effectiveness of PCV7 (*4*)<br>Monitoring incidence of non-vaccine serotypes (*5*)<br>Accelerated regulatory approval of PCV13 (*6*)<br>Informed ACIP recommendations for PCV13 use in immunocompromised adults and children (*7,8*)
Neisseria meningitidis	Conjugate vaccines<br>Serogroup B vaccines	Informed ACIP recommendations for children 11–18 y of age (*9–11*)<br>Informed ACIP recommendations for booster dose (*12*)<br>Documented vaccine effectiveness (*13*)<br>Informed ACIP infant meningococcal recommendations (*14*)<br>Evaluated potential effect on serogroup B disease in United States (*15*)
Haemophilus influenzae	Hib vaccine	Tracking postlicensure declines in Hib disease (*16*)<br>Tracking shift toward non-Hib disease (*16*)<br>Evaluated effect of vaccine shortages (*17–19*)
Group A *Streptococcus*	M-type vaccine (under development)	Estimated degrees of protection against severe group A streptococcal infections (*20,21*)
Group B *Streptococcus*	Trivalent vaccine (under development)	Informing development of vaccine to prevent early-onset (within 1 week of life) group B streptococcal disease (*22*)
Methicillin –resistant *Staphylococcus aureus*	*S. aureus* vaccine (under development)	Determining population groups to target (*23*)

*ACIP, Advisory Committee on Immunization Practices; Hib, *H. influenzae* type b vaccine; PCV7, 7-valent pneumococcal conjugate vaccine; PCV13, 13-valent pneumococcal conjugate vaccine.

References

1. Centers for Disease Control and Prevention. Preventing pneumococcal disease among infants and young children. MMWR Recomm Rep. 2000;49(RR09):1–35. PubMed

2. Nuorti JP, Whitney CG; Centers for Disease Control and Prevention. Prevention of pneumococcal disease among infants and children—use of 13-valent pneumococcal conjugate vaccine and 23-valent pneumococcal polysaccharide vaccine—recommendations of the Advisory Committee on Immunization Practices (ACIP). MMWR Recomm Rep. 2010;59(RR-11):1–18. PubMed

3. Pilishvili T, et al. Sustained reductions in invasive pneumococcal disease in the era of conjugate vaccine. J Infect Dis. 2010;201:32–41. PubMed

4. Whitney CG, et al. Effectiveness of seven-valent pneumococcal conjugate vaccine against invasive pneumococcal disease: a matched case-control study. Lancet. 2006;368:1495–502. PubMed

5. Moore MR, Whitney CG. Emergence of nonvaccine serotypes following introduction of pneumococcal conjugate vaccine: cause and effect? Clin Infect Dis. 2008;46:183–5 10.1086/524661. PubMed

6. FDA. Vaccines, Blood and Biologics: Prevnar 13. 2013 [cited 2013 November 4]. http://www.fda.gov/BiologicsBloodVaccines/Vaccines/ApprovedProducts/ucm201667.htm.

7. CDC. Use of 13-valent pneumococcal conjugate vaccine and 23-valent pneumococcal polysaccharide vaccine for adults with immunocompromising conditions: recommendations of the Advisory Committee on Immunization Practices (ACIP). MMWR Morb Mortal Wkly Rep. 2012;61:816–9. PubMed

8. CDC. Use of 13-valent pneumococcal conjugate vaccine and 23-valent pneumococcal polysaccharide vaccine among children aged 6–18 years with immunocompromising conditions: recommendations of the Advisory Committee on Immunization Practices (ACIP). MMWR Morb Mortal Wkly Rep. 2013;62:521–4. PubMed

9. CDC. Revised recommendations of the Advisory Committee on Immunization Practices to Vaccinate all Persons Aged 11–18 Years with Meningococcal Conjugate Vaccine. MMWR Morb Mortal Wkly Rep. 2007;56:794–5. PubMed

10. Harrison LH, et al. Risk of meningococcal infection in college students. JAMA. 1999;281:1906–10. PubMed

11. Harrison LH, et al. Invasive meningococcal disease in adolescents and young adults. JAMA. 2001;286:694–9. PubMed

12. CDC. Updated recommendations for use of meningococcal conjugate vaccines—Advisory Committee on Immunization Practices (ACIP), 2010. MMWR Morb Mortal Wkly Rep. 2011;60:72–6. PubMed

13. Macneil JR, et al. Early estimate of the effectiveness of quadrivalent meningococcal conjugate vaccine. Pediatr Infect Dis J. 2011;30:451–5. PubMed

14. CDC. Infant meningococcal vaccination: Advisory Committee on Immunization Practices (ACIP) recommendations and rationale. MMWR Morb Mortal Wkly Rep. 2013;62:52–4. PubMed

15. Cohn AC, et al. Changes in *Neisseria meningitidis* disease epidemiology in the United States, 1998–2007: implications for prevention of meningococcal disease. Clin Infect Dis. 2010;50:184–91. PubMed

16. MacNeil JR, et al. Current epidemiology and trends in invasive *Haemophilus influenzae* disease–United States, 1989–2008. Clin Infect Dis. 2011;53:1230–6. PubMed

17. Briere EC, et al. *Haemophilus influenzae* type b disease and vaccine booster dose deferral, United States, 1998–2009. Pediatrics. 2012;130:414–20. Epub 2012 Aug 6. PubMed http://dx.doi.org/10.1542/peds.2012-0266

18. CDC. Continued shortage of *Haemophilus influenzae* Type B (Hib) conjugate vaccines and potential implications for Hib surveillance–United States 2008. MMWR Morb Mortal Wkly Rep. 2008;57:1252–5. PubMed

19. Jackson ML, et al. Modeling insights into *Haemophilus influenzae* type b disease, transmission, and vaccine programs. Emerg Infect Dis. 2012;18:13–20. PubMed http://dx.doi.org/10.3201/eid1801.110336

20. McNeil SA, et al. Safety and immunogenicity of 26-valent group a streptococcus vaccine in healthy adult volunteers. Clin Infect Dis. 2005;41:1114–22. Epub 2005 Sep 12. PubMed

21. O'Loughlin RE, et al. The epidemiology of invasive group A streptococcal infection and potential vaccine implications: United States, 2000–2004. Clin Infect Dis. 2007;45:853–62. PubMed

22. Madhi SA, et al. Considerations for a phase-III trial to evaluate a group B Streptococcus polysaccharide-protein conjugate vaccine in pregnant women for the prevention of early- and late-onset invasive disease in young-infants. Vaccine. 2013;31(Suppl 4):D52–7. PubMed http://dx.doi.org/10.1016/j.vaccine.2013.02.029

23. Lucero CA, et al. Evaluating the potential public health impact of a *Staphylococcus* aureus vaccine through use of population-based surveillance for invasive methicillin-resistant *S. aureus* disease in the United States. Vaccine. 2009;27:5061–8. PubMed
